# Efficacy and safety of intracoronary pro-urokinase combined with low-pressure balloon pre-dilatation during percutaneous coronary intervention in patients with anterior ST-segment elevation myocardial infarction

**DOI:** 10.1186/s13019-024-02699-7

**Published:** 2024-04-05

**Authors:** Shicheng Yu, Haoxuan Jia, Shengkai Ding, Mengda Zhang, Fengyun Li, Pan Xu, Yuan Tian, Lingling Ma, Lijie Gong, Jun Feng, Zhaojin Sun, Fudong Qian, Hui Li

**Affiliations:** 1 Department of Cardiology, Lu’an Hospital of Anhui Medical University, Lu’an, Anhui 237000 People’s Republic of China; 2 Graduate School of Bengbu Medical College, Bengbu, Anhui 233004 People’s Republic of China

**Keywords:** STEMI, PCI, Pro-urokinase, Efficacy, Safety

## Abstract

**Background:**

The efficacy and safety of low-pressure balloon pre-dilatation before intracoronary pro-urokinase (pro-UK) in preventing no-reflow during percutaneous coronary intervention (PCI) remains unknown. This study aimed to evaluate the clinical outcomes of intracoronary pro-UK combined with low-pressure balloon pre-dilatation in patients with anterior ST-segment-elevation myocardial infarction (STEMI).

**Methods:**

This was a randomized, single-blind, investigator-initiated trial that included 179 patients diagnosed with acute anterior STEMI. All patients were eligible for PCI and were randomized into two groups: intracoronary pro-UK combined with (ICPpD group, *n* = 90) or without (ICP group, *n* = 89) low-pressure balloon pre-dilatation. The main efficacy endpoint was complete epicardial and myocardial reperfusion. The safety endpoints were major adverse cardiovascular events (MACEs), which were analyzed at 12 months follow-up.

**Results:**

Patients in the ICPpD group presented significantly higher TIMI myocardial perfusion grade 3 (TMPG3) compared to those in the ICP group (77.78% versus 68.54%, *P* = 0.013), and STR ≥ 70% after PCI 30 min (34.44% versus 26.97%, *P* = 0.047) or after PCI 90 min (40.0% versus 31.46%, *P* = 0.044). MACEs occurred in 23 patients (25.56%) in the ICPpD group and in 32 patients (35.96%) in the ICP group. There was no difference in hemorrhagic complications during hospitalization between the groups.

**Conclusion:**

Patients with acute anterior STEMI presented more complete epicardial and myocardial reperfusion with adjunctive low-pressure balloon pre-dilatation before intracoronary pro-UK during PCI.

**Trial registration:**

2019xkj213.

## Background

Over the past decade, patient admission due to ST-segment elevation myocardial infarction (STEMI) in China has increased nearly four-fold due to an aging population and changing lifestyles, with a pre-hospital mortality rate of up to 10% [1, 2]. Revascularization of occluded coronary arteries by primary percutaneous coronary intervention (PCI) is the evidence-based standard of therapy for patients with acute STEMI [3]. However, studies have shown that myocardial perfusion is compromised at the cellular level in nearly 50% of STEMI patients, despite the ability of PCI to restore better epicardial blood flow [4]. Suboptimal myocardial blood flow after primary PCI may be due to a distal thrombo-embolism that impairs microvascular perfusion and thus adds to infarct size, a phenomenon that is particularly pronounced in patients with a high thrombotic burden [5–8]. On the other hand, this unsatisfactory reperfusion may also be due to microvascular occlusion, vasospasm, interstitial edema, and cellular injury [9, 10].

Several studies have explored the potential use of adjunctive intracoronary fibrinolytic therapy to improve myocardial perfusion during primary PCI [11–13]. Recombinant human pro-urokinase (pro-UK) is structurally similar to alteplase and converts to active urokinase on the surface of the thrombus, producing a thrombolytic effect [14–16]. Pro-UK has been widely used as an adjunctive intracoronary fibrinolytic agent in patients with STEMI during primary PCI to improve myocardial reperfusion and clinical outcomes in chest pain centers across China. However, to our knowledge, the results have been highly discrepant, and there are reported differences in the timing and methods of intracoronary administration of pro-UK. In particular, there are no clear findings on whether to use a low-pressure balloon for pre-dilatation prior to injection.

The aim of this study was to investigate the efficacy and safety of intracoronary administration of pro-UK combined with low-pressure balloon pre-dilatation versus direct intracoronary pro-UK on myocardial reperfusion in patients with anterior STEMI undergoing PCI. Abrupt restoration of intracoronary pressure and flow triggered by PCI may play an important role in the development of reperfusion-related microvascular damage, including intramyocardial edema and hemorrhage in STEMI. Gradual reopening of the occluded infarct-related artery may limit microvascular injury in this setting. We hypothesized that adjunctive low-pressure balloon pre-dilatation before intracoronary pro-UK during PCI would reduce no-reflow and ischemia–reperfusion injury in STEMI patients compared to administration of intracoronary pro-UK directly.

## Methods

### Trial design and oversight

**Fig. 1:**
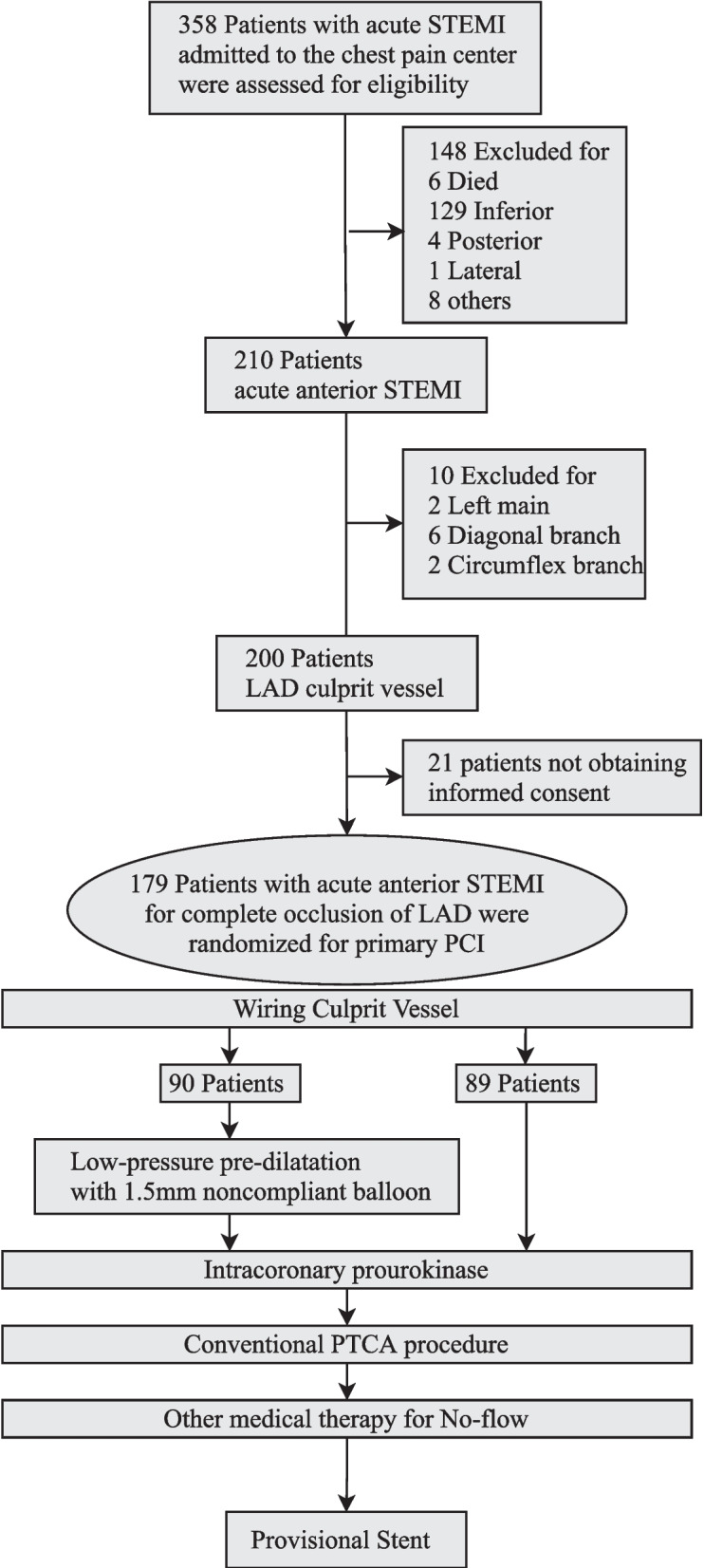
Flow chart of the trial

This single-center, randomized, single-blind, investigator-initiated trial was designed to compare the efficacy of intracoronary pro-UK combined with low-pressure balloon pre-dilatation versus direct intracoronary pro-UK administration on epicardial/myocardial reperfusion and clinical outcomes in anterior STEMI patients undergoing primary PCI (Fig. 1). The trial was funded by the Scientific Research Project of Anhui Medical University. The trial protocol was designed and approved by the ethics committee of Anhui Medical University. The Chest Pain Center in Lu'an Hospital of Anhui Medical University provided all the support needed to conduct this clinical trial, including project coordination, medical review, data management, site monitoring, and statistical oversight and analysis. The trial medication was provided by Tasly Pharmaceutical CO., LTD (China), who played no role in the trial design, conduction, or in preparation or review of the manuscript. Operative procedures in the trial were executed by experienced interventional cardiologists. An independent committee composed of cardiologists, who were blinded to trial group assignment, was responsible for adjudicating the potential trial endpoint events. In addition, the trial was overseen by a data and safety monitoring committee comprised of independent experts to ensure the accuracy of the data.

### Participant enrollment

Adult patients presenting to our center between January, 2020, and February, 2022, within 12 h of symptom onset who were scheduled for percutaneous revascularization were eligible for this study. The inclusion criteria were as follows: 1) ≥ 18 years old; 2) electrocardiographic criteria for acute anterior STEMI (≥ 2 mm in two contiguous precordial leads); and 3) provided informed consent to participate in this study. Patients were excluded if they had one of the following characteristics: 1) cardiogenic shock; 2) acute infection; 3) medical history of severe hepatic or severe renal failure (plasma creatine levels > 3 mg/dl); 4) medical history of clinically significant non-transient hematologic abnormalities; 5) current or planned long-term systemic glucocorticoid therapy; 6) history of clinically significant sensitivity to iodinated contrast agents; 7) ongoing chronic inflammatory diseases, or 8) autoimmune or malignant diseases. Prior to enrollment, all patients were fully informed of the trial design and written informed consent was required from all trial participants. Post-operative follow-up was performed at one and three months after randomization and every three months thereafter.

### Randomization and intervention

All patients who were eligible for PCI were randomized into two groups at a 1:1 ratio according to the random number principle: intracoronary pro-UK combined with (ICPpD group, *n* = 90) or without (ICP group, *n* = 89) low-pressure balloon pre-dilatation during PCI (1.5*15 mm compliant balloon, 8 atm, Medtronic). Patients received intracoronary pro-UK at a dose of 20 mg. All enrolled patients received 300 mg of aspirin, as well as a loading dose of adenosine diphosphate receptor antagonist (180 mg ticagrelor or 300–600 mg clopidogrel) and 20 mg atorvastatin in the emergency room or in the ambulance. Routine ECG testing was performed every 30 min after PCI. Drug-eluting or drug-coated stents were routinely implanted during PCI. Glycoprotein IIb/IIIa inhibitors were not allowed for any patient prior to the PCI procedure, but were allowed at the discretion of the investigator during or after the catheterization procedure.

### Efficacy endpoints and safety endpoints

The primary efficacy endpoint was defined as a composite of complete epicardial and myocardial reperfusion after PCI, defined as TIMI flow grade (TFG) 3 for epicardial reperfusion, TIMI myocardial perfusion grade (TMPG) 3 for myocardial reperfusion, and ST-segment resolution (STR) ≥ 70% at 30 min and 90 min after PCI. The secondary endpoints consisted of left ventricular (LV) function assessed by echocardiography and hemorrhagic complications before discharge. Additional prespecified exploratory end points were major adverse cardiovascular events (MACEs), which is a composite of cardiac death, recurrent myocardial infarction, heart failure, and stroke within 12 months of follow-up. Safety endpoints were defined as all-cause death, recurrent myocardial infarction, heart failure, and stroke during the follow-up time. The main safety end point was the incidence of hemorrhagic complications. Bleeding complications were classified using the GUSTO (Global Utilization of Streptokinase and Tissue Plasminogen Activator for Occluded Coronary Arteries) severity criteria [17]. All source documents were reviewed for accuracy and completeness by an independent study supervisor in the field. Endpoint events were verified by a blinded review committee.

### Measurement of epicardial and myocardial reperfusion and LV function

Coronary angiography was assessed in an independent core laboratory by experienced investigators who were blinded to treatment assignments or clinical outcomes. TFG and corrected TIMI frames of epicardial artery flow were assessed using previously described methods [18, 19]. Myocardial perfusion was assessed using TMPG and TIMI myocardial perfusion frames [20, 21], which is a new method recently described by our team for standardizing and quantifying myocardial perfusion levels [21, 22]. Routine ECGs were obtained at admission and at 30 min and 90 min after the procedure. In addition, the sum of ST-segment elevations at 20 ms after the J-point was measured in an independent laboratory. The percent resolution was categorized based on Schroder’s method as complete (≥ 70%), partial (30% to < 70%), or none (< 30%) [23]. In this study, the patient had to meet all of the mentioned four criteria to achieve the primary endpoint.

Transthoracic echocardiography and B-type natriuretic peptide (BNP) were examined in the hospital before discharge. Quantitative echocardiographic analysis of LV ejection fraction (LVEF) and other echocardiographic parameters were stored digitally for subsequent offline analysis.

### Sample size calculation

The sample size of this study was estimated based on previous similar studies using intracoronary thrombolysis during PCI in STEMI [24–28] and the follow-up information provided by the studies in our center. According to the previous research and the results of a preliminary experiment at our center, the composite endpoint (defined as TIMI flow grade (TFG) 3 for epicardial reperfusion, TIMI myocardial perfusion grade (TMPG) 3 for myocardial reperfusion, and ST-segment resolution (STR) ≥ 70% at 30 min and 90 min after PCI.) achieved immediately after PCI was 83% in the ICPpD group and 64% in the ICP group, respectively. Sample size calculations assume 80% power and a 5% (two-sided) significance level. To secure adequate power of the trial and account for an estimated 10% withdraw rate, a sample size of 90 participants (per group) was needed to test the superiority hypothesis of the study.

### Statistical analysis

Primary analysis was performed on the full analysis set on an intention-to-treat basis. The distribution of all continuous variables was assessed for normality using the Shapiro–Wilk test and is presented as mean ± standard deviation (SD) or median (Q1, Q3), appropriately. For nominal variables, either the Chi-square test or Fisher’s exact test was used to compare the groups. For continuous variables, a one-way analysis of variance (ANOVA) was performed to compare the groups. Two-sided *P*-values less than 0.05 were considered statistically significant. Survival analysis was determined using Kaplan–Meier curves followed by a log-rank test. All data analyses were conducted using GraphPad Prism version 9 for Windows.

## Results

### Relevant clinical characteristics at baseline

A total of 358 patients with acute STEMI were admitted from January 1, 2020, to February 28, 2022, at our center. Initially, 148 patients were excluded because of non-anterior-wall infarction or death before catheterization could be performed. Of the remaining 210 patients, 10 had acute anterior STEMI due to occlusion of the left main coronary artery, diagonal branches, or circumflex branches and were further excluded. Another 21 patients were excluded because they did not provide informed consent. Finally, 90 patients were enrolled in the ICPpD group and 89 patients were enrolled in the ICP group.

The clinical characteristics of patients in the two groups are described in Table 1. The median age of the study population was 62.2 (62.2 ± 13.0) years; 61.9 ± 13.2 years for the ICP group and 62.5 ± 12.8 years for the ICPpD group. Male patients comprised 72.1% of the total cohort, and 45.3% of the cohort had hypertension and 11.7% had diabetes mellitus. The two groups had a similar hemodynamic status, as determined by blood pressure, heart rate, and Killip class. No significant differences were observed between the two groups for any of the above indicators.

**Table 1:** Baseline clinical and treatment characteristics of the randomized participants (*n* = 179)

Baseline Characteristics	Total (*n* = 179)	ICP group (*n* = 89)	ICPpD group (*n* = 90)	*P* Value
**Demographics**				
Age, mean (SD), y	62.2 ± 13.0	61.9 ± 13.2	62.5 ± 12.8	0.766
Male	129(72.1)	62(69.7)	67(74.4)	0.173
Female	50(27.9)	27(30.3)	23(25.6)	0.283
BMI, mean (SD), kg/m^2^	24.98(3.07)	24.26(3.31)	25.57(3.82)	0.124
Weight, mean (SD), kg	68.99 ± 10.89	67.91 ± 12.21	70.32 ± 8.10	0.349
**Presenting characteristics, mean (SD)**				
Heart rate, beat/min	82.56 ± 15.52	75.4 ± 12.54	91.5 ± 15.503	0.128
Systolic blood pressure, mmHg	123.7 ± 22.84	120 ± 27.009	128.25 ± 19.19	0.624
**Medical history**				
Hypertension	81 (45.3)	38 (42.7)	43 (47.8)	0.531
Hypercholesterolemia	51 (28.5)	27 (30.3)	24 (26.7)	0.323
Diabetes mellitus	21 (11.7)	10 (11.2)	11 (12.2)	0.317
Atrial fibrillation	11 (6.2)	6 (6.7)	5 (5.6)	0.673
Renal impairment	7 (3.9)	3 (3.4)	4 (4.4)	0.783
**Smoking**				
Current	67 (37.4)	35 (39.3)	32 (35.6)	0.364
Former (stopped > 3 mo)	16 (39.3)	9 (10.1)	7 (7.8)	0.247
Never	96 (53.6)	45 (50.6)	51 (56.7)	0.155
**Preexisting maintenance medication**				
Aspirin	32 (17.9)	15 (16.9)	17 (18.9)	0.754
Statin	60 (33.5)	31 (34.8)	29 (32.2)	0.813
β-blocker	66 (36.9)	34 (38.2)	32 (35.6)	0.471
ACE inhibitor/ARB	54 (30.2)	25 (28.1)	29 (32.2)	0.542
**Killip class**				
I	158 (88.3)	77 (86.5)	81 (90.0)	0.267
II‒IV	21 (11.7)	12 (13.5)	9 (10.0)	0.361
**TIMI risk score**				
Low risk (0–3)	32 (17.9)	15 (16.9)	17 (18.9)	0.383
Moderate risk (4–6)	115 (64.3)	56 (62.9)	59 (65.6)	0.186
High risk (7–14)	32 (17.9)	18 (20.2)	14 (15.6)	0.369
**Initial blood results on admission**				
Glucose, mean (SD), mmol/L	7.50 ± 3.19	7.73 ± 3.46	7.27 ± 2.90	0.343
Creatinine, mean (SD), μmol/L	75.71 ± 25.53	75.25 ± 26.29	76.17 ± 24.91	0.809
Uric acid, mean (SD), μmol/L	365.00 ± 110.60	355.64 ± 99.66	374.25 ± 120.362	0.262
Total cholesterol, mean (SD), mmol/L	4.981 ± 1.33	5.10 ± 1.34	4.86 ± 1.32	0.235
Triglycerides, mean (SD), mmol/L	1.84 ± 2.10	1.57 ± 1.19	2.10 ± 2.69	0.089
HDL-C, mean (SD), mmol/L	1.26 ± 0.30	1.31 ± 0.32	1.21 ± 0.28	0.022
LDL-C, mean (SD), mmol/L	3.15 ± 1.06	3.24 ± 1.11	3.07 ± 1.01	0.279
Creatine kinase isoenzyme, mean (SD), IU/L	190.60 ± 137.50	182.94 ± 138.52	198.56 ± 136.85	0.467
Troponin I, median (IQR), ng/mL	4882.00 ± 13,801.00	6562.34 ± 16,067.71	4161.18 ± 13,312.99	0.732
Platelet count, mean (SD), × 10^9^/L	215.90 ± 62.14	214.47 ± 52.02	217.35 ± 71.18	0.782
Mean platelet volume, mean (SD), fL	10.93 ± 1.23	10.85 ± 1.10	11.00 ± 1.36	0.459
Leukocyte count, mean (SD), × 10^9^/L	10.90 ± 3.28	11.00 ± 3.38	10.80 ± 3.20	0.717
Neutrophil count, mean (SD), × 10^9^/L	8.64 ± 3.25	8.76 ± 3.36	8.52 ± 3.16	0.653
Lymphocyte percentage, mean (SD), %	13.9 ± 7.1	13.9 ± 7.0	13.9 ± 7.4	0.967
Neutrophil, mean (SD), %	77.7 ± 9.4	78.1 ± 9.1	77.2 ± 9.8	0.579
Monocyte, mean (SD), %	7.7 ± 3.3	7.3 ± 3.3	8.1 ± 3.3	0.132
Eosinophil, mean (SD), %	0.7 ± 0.1	0.7 ± 1.0	0.7 ± 1.1	0.703
Basophil, mean (SD), %	0.2 ± 0.1	0.2 ± 0.1	0.2 ± 0.2	0.309

*ICPpD group* Intracoronary pro-UK combined with pre-dilatation, *ICP group* Intracoronary pro-UK

### Time intervals in chest pain center and procedure characteristics

Detailed data regarding time intervals in the chest pain center are shown in Table 2. There were no differences between the ICP and ICPpD groups for symptom onset to first medical contact (FMC), arterial sheath insertion, randomization by CAG, low-pressure balloon pre-dilatation, intracoronary pro-UK, conventional PTCA, and provisional stent. Similar symptom onset to FMC and randomization intervals were observed between the two groups. The median time from wiring of culprit vessel to intracoronary pro-UK was 3.5 (2.2–4.3) min in the ICP group and 3.8 (2.0–4.0) min in the ICPpD group (*P* = 0.691)*.* During the procedure, patients in both groups received a loading dose of 300 mg of aspirin and 180 mg of ticagrelor (Table 3). A similar rate of radial access was observed in both the ICP (92.1%) and ICPpD (91.1%) groups, and 30.3% and 34.5% of patients in the ICP and ICPpD groups, respectively, had single vessel involvement (*P* = 0.588). All patients in this study accepted 20 mg intracoronary pro-UK according to the trial protocol, and the rate of PCI with stent implant was 99.3% and 98.5% in the ICP and ICPpD groups, respectively (*P* = 0.922).

**Table 2:** Time intervals recorded in the chest pain center

Time Delay (min)	Intracoronary pro-UK (*n* = 89)	Intracoronary pro-UK combined with pre-dilatation (*n* = 90)	*P* Value
Symptom onset to first medical contact	205 (115–399)	213 (109–411)	0.718
Symptom onset to arterial sheath insertion	246 (154–441)	247 (144–440)	0.904
Symptom onset to randomization by CAG	249 (158–447)	251 (148–445)	0.866
Symptom onset to wiring of culprit vessel	255 (162–459)	257 (155–452)	0.797
Symptom onset to low-pressure balloon pre-dilatation	-	258 (158–459)	-
Symptom onset to intracoronary pro-urokinase	257 (166–463)	261 (161–462)	0.823
Symptom onset to conventional PTCA	261 (170–467)	264 (164–465)	0.742
Symptom onset to provisional stent	266 (174–472)	271 (169–472)	0.831
Interval between wiring of culprit vessel and intracoronary pro-urokinase, median (IQR), min	3.5 (2.2–4.3)	3.8 (2.0–4.0)	0.691

**Table 3:** Procedure characteristics

	**Intracoronary pro-UK No. (%) of patients (*n* = 89)**	**Intracoronary pro-UK combined with pre-dilatation No. (%) of patients (*n* = 90)**	***P* Value**
**Acute therapy following the first medical contact, No./total (%)**			
Loading dose of aspirin, 300 mg	89 (100)	90 (100)	0.977
Loading dose of ticagrelor, 180 mg	89 (100)	90 (100)	0.977
Inhaled oxygen	74 (83.2)	77 (85.6)	0.893
Percutaneous morphine	9 (10.1)	10 (11.1)	0.615
**Artery access**			
Right radial artery	79 (88.8)	77 (85.6)	0.733
Left radial artery	3 (3.4)	5 (5.6)	0.524
Right femoral artery	7 (7.9)	8 (8.9)	0.658
**Coronary artery disease**			
Single vessel disease	27 (30.3)	31 (34.5)	0.588
Multivessel disease	62 (69.7)	59 (65.6)	0.636
**Intracoronary pro-urokinase, No./total (%)**			
20 mg according to protocol	89 (100)	90 (100)	0.977
Duration of infusion, mean (SD), min	3.4 (1.9)	3.6 (1.6)	0.856
**Stent implantation**			
PCI with stent implant	87 (99.3)	88 (98.5)	0.922
Total No. of stents deployed, mean (SD)	2.14 (1.1)	2.27 (1.1)	0.848
Total length of stents deployed, mean (SD), mm	29.52 (11.7)	31.73 (10.6)	0.632
Poststent dilatation	11 (12.4)	13 (14.4)	0.509
Intravenous glycoprotein IIb/IIIa antagonist after PCI	81 (91.1)	83 (92.2)	0.724

### Low-pressure balloon pre-dilatation improved reperfusion and cardiac function

**Fig. 2:**
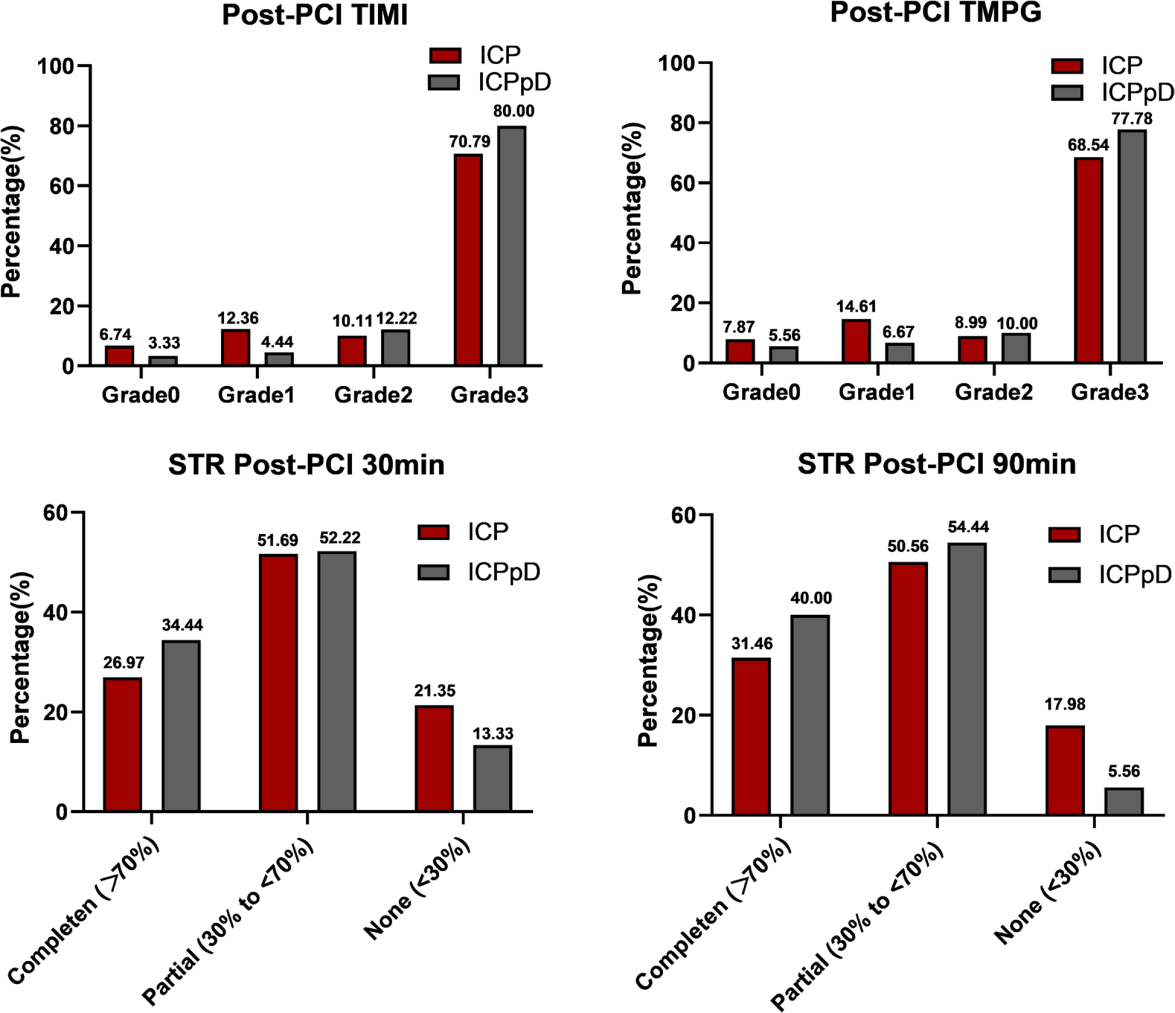
Individual components of the primary end point in the two treatment arms

In our study, full epicardial and myocardial reperfusion was defined as TIMI 3 and TMPG 3, with complete STR post-PCI at 30 min and 90 min. Complete epicardial and myocardial reperfusion was achieved by 26.2% of patients in the ICPpD group that compared to 14.8% of patients in the ICP arm (Table 4). During PCI, intracoronary pro-UK combined with low-pressure balloon pre-dilatation showed significant differences in the incidence of each component of the primary endpoint (Fig. 2). Briefly, TIMI flow grade (80.0% versus 70.8%, *P* = 0.017) and TMPG 3 (77.8% versus 68.5%, *P* = 0.013) were higher in the ICPpD group after PCI compared to the ICP group. The percentage of patients with STR ≥ 70% at 30 min after PCI and STR ≥ 70% at 90 min after PCI (31.5% versus 40.0%, *P* = 0.044) was significantly higher in the ICPpD group (27.0% versus 34.4%, *P* = 0.047). However, there was no difference in thrombus burden at initial angiography between the two groups, shown as the TIMI thrombotic grade.

**Table 4:** Procedure outcomes

	**Intracoronary pro-UK No. (%) of patients (*n* = 89)**	**Intracoronary pro-UK combined with pre-dilatation No. (%) of patients (*n* = 90)**	***P* Value**
**TIMI thrombus grade at initial angiography**			
0 (no thrombus)	8 (8.99)	8 (8.89)	0.927
1–2 (definite, < ½ vessel diameter)	13 (14.6)	12 (13.3)	0.579
3 (3, definite, > ½ but < 2 vessel diameters)	17 (19.1)	19 (21.1)	0.642
4 (definite thrombus ≥ 2 vessel diameters)	5 (5.6)	7 (7.8)	0.769
5 (total occlusion)	46 (51.7)	44 (48.9)	0.875
**TIMI flow grade at initial angiography**			
0 (no flow)	61 (68.5)	61 (67.8)	0.631
1 (minimal flow)	12 (13.5)	14 (15.6)	0.611
2 (slow but complete)	9 (10.1)	8 (8.9)	0.528
3 (normal flow)	7 (7.87)	7(7.78)	0.827
**TIMI flow grade after conventional PTCA procedure**			
0 (no flow)	6 (6.7)	3 (3.3)	0.031
1 (minimal flow)	11 (12.4)	4 (4.4)	0.021
2 (slow but complete)	9 (10.1)	11 (12.2)	0.059
3 (normal flow)	63 (70.8)	72 (80.0)	0.017
**TMPG flow after PCI**			
0	7 (7.9)	5 (5.6)	0.055
1	13 (14.6)	6 (6.7)	0.027
2	8 (9.0)	9 (10.0)	0.056
3	61 (68.5)	70 (77.8)	0.013
**Corrected TIMI frame count (CTFC)**	27.42 ± 5.457	23.17 ± 6.328	0.036
**Reperfusion syndrome**			
Premature ventricular contraction	24 (27.0)	16 (17.8)	0.031
Ventricular tachycardia	9 (10.1)	7 (7.8)	0.057
Ventricular fibrillation	1 (1.1)	2 (2.2)	0.252
Bradycardia	1 (1.1)	1 (1.1)	0.881
Atrioventricular block	0 (0)	0 (0)	-
Transient drop of blood pressure ≥ 30 mmHg	11 (12.4)	8 (8.9)	0.728
Total	43 (48.3)	34 (37.8)	0.022
**Necessity of other medical therapy for No-flow**			
Nitroglycerin	29 (32.6)	17 (18.9)	0.028
Sodium nitroprusside	13 (14.6)	11 (12.2)	0.337
Verapamil	3 (3.4)	2 (2.2)	0.835
Adenosine	4 (4.5)	6 (6.7)	0.649
Aspiration thrombectomy	9 (10.1)	7 (7.8)	0.753
Total	58 (65.2)	43 (47.8)	0.018
**Resolution of ST-segment elevation**			
30 min after PCI			
Complete (> 70%)	24 (27.0)	31 (34.4)	0.047
Partial (30% to < 70%)	46 (51.7)	47 (52.2)	0.872
None (< 30%)	19 (21.4)	12 (13.3)	0.034
90 min after PCI			
Complete (> 70%)	28 (31.5)	36 (40.0)	0.044
Partial (30% to < 70%)	45 (50.6)	49 (54.4)	0.896
None (< 30%)	16 (18.0)	5 (5.6)	0.026
**Thrombosis and coagulation indexes**			
Prothrombin time (PT), sec	12.7 ± 2.979	12.4 ± 1.733	0.546
International Normalized Ratio (INR)	1.03 ± 0.317	0.99 ± 0.154	0.515
D-Dimer, mg/L	1.71 ± 4.238	1.47 ± 1.404	0.798
Fibrinogen degradation products (FDP), mg/L	3.23 ± 2.777	5.74 ± 4.629	0.069
MPO-DNA, ng/mL	925.67 ± 93.23	938.88 ± 71.32	0.916
**Bleeding events in hospital**			
Gingival	9 (10.1)	7 (7.8)	0.925
Hematuria	2 (2.3)	4 (4.5)	0.873
Hemoptysis	0 (0)	0 (0)	-
Gastrointestinal hemorrhage	2 (2.3)	2 (2.2)	0.912
Cath access site bleeding	4 (4.5)	3 (3.3)	0.892
Intraocular hemorrhage	1 (1.1)	0 (0)	-
Epistaxis	3 (3.5)	4 (4.5)	0.851
Others	1 (1.1)	0 (0)	-
Total, *n* (%)	22 (24.7)	20 (22.2)	0.794

Furthermore, LVEF before discharge was higher in the ICPpD group compared to the ICP group (54.0 ± 9.6% versus 49.0 ± 8.3%, *P* = 0.023). Echocardiography indices, such as LV end-diastolic diameter (LVDd), LV end-diastolic volume, and pulmonary artery systolic pressure (PASP), were better in the ICPpD group (Table 5). Furthermore, B-type natriuretic peptides (BNP) was significantly lower in the ICPpD group compared to the ICP group (290.35 ± 28.72 pg/mL versus 417.23 ± 18.81 pg/mL, *P* = 0.012).

**Table 5:** LV function before discharge

	**Intracoronary pro-UK (*n* = 89)**	**Intracoronary pro-UK combined with pre-dilatation (*n* = 90)**	***P* Value**
**Echocardiography**			
Left ventricular end-diastolic diameter (LVDd), mm	53.37 ± 7.99	48.26 ± 4.23	0.039
left ventricular end-diastolic volume, ml	127.18 ± 11.98	119.09 ± 12.67	0.027
Interventricular Septum Thickness (IVSTH), mm	10.06 ± 2.00	9.61 ± 1.66	0.108
Posterior wall thickness (PWTH), mm	10.02 ± 1.48	9.76 ± 1.36	0.241
Left ventricular ejection fraction (LVEF), %	49.0 ± 8.3	54.0 ± 9.6	0.023
Stroke volume (SV), ml	68.85 ± 15.25	66.5 ± 13.7	0.517
Left atrial diameter (LAD), mm	38.03 ± 5.09	39.00 ± 5.13	0.216
Pulmonary artery systolic pressure (PASP), mmHg	39.07 ± 9.52	30.97 ± 9.02	0.046
**Biomarker**			
B-type natriuretic peptides (BNP), pg/ml	417.23 ± 18.81	290.35 ± 28.72	0.012

### Clinical and safety outcomes

**Fig. 3:**
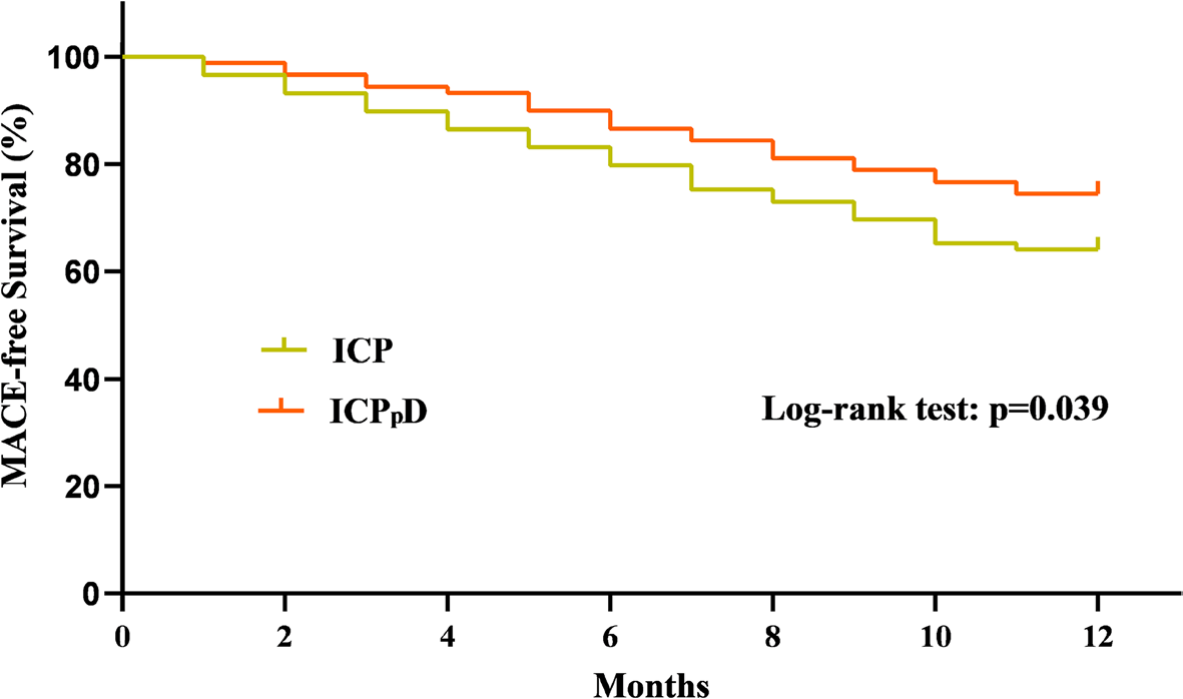
Kaplan–Meier curves for MACE during the 12-month follow-up of patients in the ICP and ICPpD groups. Log-rank test, *P* = 0.039. MACE major adverse cardiovascular events, including cardiac death, reinfarction, heart failure, and stroke

At the 12-month follow-up, the incidence of composite clinical endpoint (death, recurrent myocardial infarction, heart failure, and stroke) was markedly lower in the ICPpD group compared to the ICP group (25.6% versus 36.0%, *P* = 0.036), and the rate of heart failure was significantly lower (30.3% versus 21.1%, *P* = 0.039) (Table 6). The 12-month event-free survival rate was significantly higher in the ICPpD group compared to the ICP group (Fig. 3, by log-rank test, *P* = 0.039). Clinical and safety outcomes at the 12-month follow-up are shown in Table 6. The rate of minor non-ICH bleeding was similar (15.7% versus 12.2%, *P* = 0.646) between groups. There was one major non-ICH bleed in the ICP group (1.1%) compared to none in the ICPpD group (0%).

**Table 6:** Clinical and safety outcomes

	**Total No. (%) of patients**	**Intracoronary pro-UK No. (%) of patients**	**Intracoronary pro-UK combined with pre-dilatation No. (%) of patients**	***P* Value**
**Clinical outcomes**				
Cardiac death	5 (2.8)	3 (3.4)	2 (2.2)	0.923
Reinfarction	4 (2.2)	2 (2.3)	2 (2.2)	0.947
Heart failure	46 (25.7)	27 (30.3)	19 (21.1)	0.039
Stroke	0 (0)	0 (0)	0 (0)	—
Combined clinical outcome	55 (30.7)	32 (36.0)	23 (25.6)	0.036
**Safety outcomes**				
Minor non-ICH bleeding	25 (14.0)	14 (15.7)	11 (12.2)	0.646
Major non-ICH bleeding	1 (0.6)	1 (1.1)	0 (0)	—
ICH bleeding	0 (0)	0 (0)	0 (0)	—

## Discussion

This was a randomized, single-blind, investigator-initiated trial that studied the effects of adjunctive low-pressure balloon pre-dilatation before intracoronary pro-UK during PCI among patients with acute anterior STEMI presenting within 12 h of symptoms onset. We found that adjunctive low-pressure balloon pre-dilatation provided more comprehensive epicardial and myocardial reperfusion compared with intracoronary pro-UK directly. The efficacy outcomes showed that cardiac function and BNP were improved in the ICPpD group compared with the ICP group during hospitalization and at 12 months follow-up. The clinical and safety outcomes indicated that the incidence of MACEs, especially heart failure hospitalizations, was lower in the ICPpD group compared to the ICP group at 12 months follow-up. Furthermore, intracoronary pro-UK combined with low-pressure balloon pre-dilatation did not increase the rates of hemorrhagic complications. Our data suggest that adjunctive low-pressure balloon pre-dilatation may be a useful and safe method during PCI to improve the efficacy of intracoronary pro-UK by reducing no-reflow and ischemia–reperfusion injury.

PCI has been considered the most effective therapy for acute STEMI for reducing myocardial infarct size and preserving LV systolic function [3, 6]. However, mortality and morbidity remain high after treatment. Timely and complete reperfusion is the most effective way to limit infarct size and subsequent ventricular remodeling, but reperfusion of the myocardium and coronary circulation itself can also lead to irreversible myocardial damage, which contributes to the final infarct size [29, 30]. It has been widely accepted that microvascular obstruction is an irreversible form of myocardial reperfusion injury [31], which may be due to external compression of capillaries by swollen endothelial cells and cardiomyocytes, microembolization of friable material released by atherosclerotic plaques, platelet microthrombi, and blockage by neutrophils [32–34]. Therefore, thrombolytic therapy combined with PCI has the potential to reduce reperfusion injury, theoretically. Previous studies demonstrated that intracoronary pro-UK injection may increase TIMI grades and TMPG [26]. However, the role of intracoronary injection of pro-UK during PCI remains controversial due to the timing and mode of administration and the diversity of patients, and low-pressure balloon pre-dilation before intracoronary injection of pro-UK might be of benefit to patients with STEMI, in view of the observation that gradual reopening of the occluded infarct-related artery led to better-preserved coronary microvascular integrity and smaller myocardial infarction size.

In our study, all patients enrolled had acute anterior STEMI due to left anterior descending coronary artery occlusion. This facilitates a more accurate assessment of the differences in the effects of different reperfusion strategies on cardiac function in the short- to mid-term follow-up. In this context, the correlation between impaired cardiac function and no-reflow or ischemia–reperfusion injury can avoid interference by other factors. Arterial thrombosis results from the interaction of pro- and anti-thrombotic forces in the blood, which can be reversed or enhanced by a variety of factors [35, 36]. In the present study, mechanical forces generated by low-pressure balloon dilation could have promoted the fragmentation and dissolution of early unstable thrombi and played a fulcrum role in reversing thrombus propagation and initiating the thrombolytic process. In addition, low-pressure balloon pre-dilatation can create a microchannel within the thrombus, allowing antithrombotic factors and thrombolytic agents in the blood to penetrate into the distal segment of the occluded coronary artery [37, 38], increasing the contact area and time of thrombolytic drugs at the thrombus. At the same time, unlike direct intracoronary pro-UK injection, low-pressure pre-dilatation of small balloons can prevent the formation of larger emboli while fragmenting the thrombus, reducing emboli and microvascular obstruction of the distal coronary artery. This may be responsible for better clinical and safety outcomes. These can be evidenced by the better cardiac function and lower BNP in the ICPpD group. On the other hand, microchannels formed within the thrombus by low-pressure balloon pre-dilatation can provide low-flow blood to the ischemic/infarcted region prior to non-pre-dilation procedures. Compared with non-pre-dilation procedures, the pre-dilation approach can reduce ischemia–reperfusion injury to cardiomyocytes. In this study, we found that the incidence of reperfusion syndrome (defined as frequent premature ventricular beats, ventricular tachycardia/fibrillation, bradycardia, AV block, and transient drop in blood pressure ≥ 30 mmHg) was significantly lower in the ICPpD group than in the ICP group (37.8% versus 48.3%,* P* = 0.022). These results suggest that pre-dilatation is beneficial for myocardial protection. The underlying mechanism may be attributed to the fact that initial canalization by low-pressure balloon pre-dilatation allows mild reperfusion of distal segments, enabling paralyzed smooth muscle cells to be resuscitated to counteract the pressure burden from subsequent full reperfusion. A previous study also suggested that, compared to abrupt reperfusion, pressure-controlled reperfusion of the culprit vessel by means of through gradual reopening of the occluded infarct-related artery led to better-preserved coronary microvascular integrity and smaller myocardial infarction size [39]. Therefore, our data suggest that low-pressure balloon pre-dilatation before intracoronary injection of pro-UK could provide more complete coronary revascularization and better cardiac function, improving patient prognosis with reliable clinical safety.

There are some limitations in our study. First, the 12 months follow-up time was relatively short to observe the long-term efficacy and safety of intracoronary pro-UK combined with low-pressure balloon pre-dilatation during PCI in STEMI patients. Secondly, the relatively small sample size and the non-blinded design may lead to the presence of statistical and observer bias. Also, the fixed dose of pro-UK per administration (20 mg) was not adjusted according to individual patient body weight. Taking into consideration that different drug dosages may influence efficacy and safety, a more appropriate pro-UK dosage should be explored in future studies. In addition, microvascular obstruction and ischemia–reperfusion injury were indirectly evaluated by measuring cardiac function. Finally, adjunct thrombolysis to primary PCI is common in China, but it is not a routine procedure in other countries, regions, or continents. A more precise assessment of the amount of microvascular obstruction using gadolinium-enhanced magnetic resonance image is necessary in future studies.

## Conclusion

In patients with acute anterior STEMI presenting within 12 h of symptom onset, adjunctive low-pressure balloon pre-dilatation prior to intracoronary pro-urokinase during PCI could provide more comprehensive epicardial and myocardial reperfusion compared with intracoronary pro-urokinase alone.

## Data Availability

The raw data supporting the conclusions of this article will be made available by the corresponding author to any qualified researcher.

## Abbreviations

pro-UK: Pro-urokinase
PCI: Percutaneous coronary intervention
MACEs: Major adverse cardiovascular events
TMPG3: TIMI myocardial perfusion grade 3
STEMI: ST-segment elevation myocardial infarction
PCI: Percutaneous coronary intervention
TFG: TIMI flow grade
TMPG: TIMI myocardial perfusion grade
CTFC: Corrected TIMI frame count
STR: ST-segment resolution
LV: Left ventricular
LVEF: LV ejection fraction
SD: Standard deviation
ANOVA: Analysis of variance
